# Biocompatibility and Antimicrobial Activity of Electrospun Fibrous Materials Based on PHB and Modified with Hemin

**DOI:** 10.3390/nano13020236

**Published:** 2023-01-05

**Authors:** Polina M. Tyubaeva, Ivetta A. Varyan, Elena D. Nikolskaya, Mariia R. Mollaeva, Nikita G. Yabbarov, Maria B. Sokol, Margarita V. Chirkina, Anatoly A. Popov

**Affiliations:** 1 Emanuel Institute of Biochemical Physics, Russian Academy of Sciences, 4 Kosygina Street, 119334 Moscow, Russia; 2 Academic Department of Innovational Materials and Technologies Chemistry, Plekhanov Russian University of Economics, 36 Stremyanny Per., 117997 Moscow, Russia

**Keywords:** poly-3-hydroxybutyrate, hemin, electrospun fibrous materials, biocompatibility, antimicrobial activity

## Abstract

The effect of the hemin (Hmi) on the structure and properties of nanocomposite electrospun materials based on poly-3-hydroxybutyrate (PHB) is discussed in the article. The additive significantly affected the morphology of fibers allowed to produce more elastic material and provided high antimicrobial activity. The article considers also the impact of the hemin on the biocompatibility of the nonwoven material based on PHB and the prospects for wound healing.

## 1. Introduction

The interest in materials from renewable resources for formulation of innovative biomedical materials is growing rapidly [1]. High attention is paid to biopolymers—for instance, to polyhydroxyalkanoates (PHA)—which is a class of sustainable aliphatic polyesters produced by various microorganisms [2]. 

**Figure 1:**
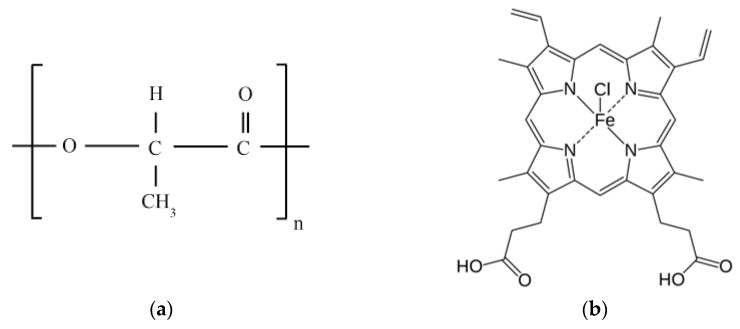
Structural formulas of PHB (**a**) and hemin (**b**).

Poly(3-hydroxybutyrate) (PHB) has become the most widespread biopolymer among all PHA due to the large number of advantages. PHB is biodegradable, biocompatible, and thermoplastic polymer [3]. Figure 1a shows the monomeric link of the PHB. PHB particles could be extracted from microorganisms, which synthesize, store, and able to degrade this polymer as a natural source of energy [4]. PHB decay products are nontoxic [4]. Moreover, PHB is able to be decomposed in a short period—Singh and coauthors reported full PHB decay during 30 days in 25% humidified compost [5]. PHB has found wide application in biomedicine [6]: scaffolds [7] and implants [8] design in tissue engineering; nanoparticles for controlled drug release [9] and delivery [10].

The PHB-based composites become popular in the biomedical application due to high biocompatibility [11,12]. Biomedical PHB-based materials with a large surface area including highly porous films [13] and fibrous materials [14] possess high similarity with the structures and surfaces of live organisms and promotes cell adhesion, viability, migration and growth [15]. PHB has shown also high efficiency in design of the new materials for wound healing [16,17].

However, industrial and commercial application of PHB-based materials is limited due to poor mechanical properties—low tensile strength and elongation [18]. Nevertheless, many collectives developed effective methods for PHB modification [19]. Especial efficacy demonstrated various nanocomposites based on PHB and biopolymers or additives of natural origin: poly(ethylene glycol) [20], polylactide [21], polycaprolactone [22], chitosan [23], nanoparticles [24], catalysts and enzymes [25], bioactive molecules [26].

A number of efforts were made by the different collectives in order to improve or endow PHB with specific properties: control of hydrophobicity [26,27] and permeability [28], increase mechanical [29] and antimicrobial [30,31] properties.

Among the number of additives, porphyrins represent a particular interest. Researchers apply widely synthetic and natural porphyrins in biomedicine [32,33], photo- and chemotherapy [34,35,36]. Most of the porphyrins are biocompatible, chemically and thermally stable [37]. Moreover, the porphyrins demonstrated a high antimicrobial and antiviral activity [38]. Earlier, the several groups developed the porphyrin-polymer systems through hydrogen bonding, weak interactions (hydrophobic or electrostatic) or coordination bounding [39,40].

The researchers display especial interest to natural porphyrins, among which is hemin (Hmi). Figure 1b shows the structural formula of Hmi. Hmi is applied in various biomedical materials: as a moiety promoting protein-polymer binding [41]; as a container for bioactive molecules [42]; as a biocatalyst [43]. The chemical structure explains a wide variety of applications and unique properties of Hmi, among which, probably the most important—biocompatibility [44] and high antimicrobial activity [45].

In this research, we formulated PHB–Hmi composites by electrospinning method (ES) [46]. ES allowed to obtain fibrous materials with a large surface area and constant distribution of Hmi in polymer matrix, which is very valuable in the production of biocompatible materials [47]. A number of reports are described application of electrospun for PHB-porphyrin composite materials fromulation: polystyrene/polyhydroxybutyrate/graphene/tetraphenylporphyrin [48], polyhydroxybutyrate/Hemin [49], polyhydroxybutyrate/tetraphenylporphyrin with Fe [50], and polyhydroxybutyrate/5,10,15,20-tetrakis(4-hydroxy-phenyl)-21H,23H-porphine [51].

In our previous study, we described the nature of Hmi effect on the supramolecular structure of PHB formation [52]. Comparing the formulated composite with the HPB fibers supplemented with a synthetic Fe^3+^ porphyrin complex, we revealed the high potential of these composite materials based on PHB–Hmi fibers [53].

The main goal of this study was assessment of the changes in the structure and properties of PHB under the influence of hemin, and influence evaluation of the Hmi molecular complexes on the biocompatibility and antimicrobial activity.

## 2. Materials and Methods

### 2.1. Materials

Polyester of natural origin—poly-3-hydroxybutyrate (PHB) was used in the work [49,53]. PHB was used in the form of a finely dispersed powder (16F series, BIOMER, Schwalbach am Taunus, Germany), characterized by 59% of crystalline phase, 206 kDa of molecular weight, 1.248 g/cm^3^ of density (Figure 1a). A tetrapyrrole complex of natural origin—hemin (Hmi) was used in the work (Figure 1b) [54]. Hmi was obtained by the extraction method from the bovine blood (production by Aldrich Sigma, Saint Louis, MO, USA). Phosphate buffered saline (PBS) (Biolot, St. Petersburg, Russia); 3-(4,5-dimethyl-2-thiazolyl)-2,5-diphenyl-2H-tetrasolium bromide (MTT) and Mowiol (Sigma-Aldrich, St. Louis, MO, USA); 96% ethanol (Chimmed, Moscow, Russia); Dulbecco’s modified Eagle’s medium (DMEM) (Gibco, Waltham, MA, USA); fetal bovine serum (FBS) (Gibco, Waltham, MA, USA); 0.9% saline (PanEco, Moscow, Russia); dimethyl sulfoxide (DMSO) (Amreso, Solon, OH, USA); 0.02% EDTA; 0.05% trypsin solutions (Gibco, Waltham, MA, USA); and gentamycin (PanEco, Moscow, Russia) were used for the experiments with cell cultures.

### 2.2. Methods

#### 2.2.1. Preparation of the Electrospun Materials

Electrospinning (ES) method was used for obtaining the fibrous materials based on PHB–Hmi [49,52]. The laboratory unit EFV-1 (Moscow, Russia) was single-capillary. Conditions of the ES process are given in the Table 1.

Since Hmi is soluble in N,N-dimethylformamide and PHB is soluble in chloroform, the method of double-solution electrospinning was used for obtaining PHB–Hmi fibers [55,56]. For preparation of forming solutions PHB powder was dissolved in chloroform at a temperature of 60 °C and Hmi powder was dissolved in N,N-dimethylformamide at a temperature of 25 °C. Both solutions were homogenized and were used 12 h after manufacture. The properties of the forming solutions based on PHB–Hmi are given in Table 2.

#### 2.2.2. Scanning Electron Microscopy

Images of electrospun materials based on PHB–Hmi were obtained by scanning electron microscopy using the Tescan VEGA3 (Brno, Czech Republic) on the samples with a platinum layer. 

#### 2.2.3. Mechanical Analysis

Tensile strength and elongation at break were obtained by the mechanical test on the tensile compression testing machine Devotrans DVT GP UG 5 (Istanbul, Turkey) on the samples 10 × 40 mm at the stretching speed was 25 mm/min without preload pressure. All data were averaged on the ten samples. Tensile strength was registered by the Devotrans software with the average statistical error in measuring thermal effects was ±0.02 MPa. Elongation at break, *ε*, was calculated as:

(1) $$\varepsilon =\frac{\Delta l}{l_{0}}\times 100\%$$

where ∆*l*—the difference between the final and initial length of the sample; *l*_0_—the initial length of the sample. The average statistical error in measuring thermal effects was ±0.2%.

#### 2.2.4. X-ray Diffraction Analysis

Degree of crystallinity of PHB and the average sizes of crystallites were obtained by X-ray diffraction analysis on the HZG4 diffractometer (Freiberger Präzisionsmechanik, Germany) HZG4 diffractometer (Freiberger Präzisionsmechanik, Germany). To calculate the degree of crystallinity, the method was used [57].

Average sizes of PHB crystallites, *L*_020_, were calculated from diffractograms obtained with the Bragg–Brentano method using the Selyakov–Scherrer formula, the method was used [58].

#### 2.2.5. Differential Scanning Calorimetry

Thermal properties of the PHB–Hmi samples were obtained by differential scanning calorimeter (DSC) using Netzsch 214 Polyma (Selb, Germany), in an argon atmosphere, with a heating rate of 10° K/min and with a cooling rate of 10° K/min with samples’ weight 6–7 mg. The DSC temperature program included 2 heating from 20 °C to 220 °C and 2 cooling to 20 °C with average statistical error 2.5%.

Enthalpy of melting, ∆*H*, was calculated by NETZSCH Proteus software according to the standard technique [59].

Crystallinity degree, *χ*, was defined from the melting peak as:

(2) $$\chi =\frac{\Delta H}{H_{PHB}}\times 100\%$$

where ∆*H*—melting enthalpy; *H_PHB_*—melting enthalpy of the ideal crystal of the PHB; 146 J/g [60]; *C*—the content of the PHB in the composition.

#### 2.2.6. Wetting Contact Angle

Wetting contact angle is a measure of wettability of the surface of the PHB–Hmi samples. Water drops (2 µL) were applied to three different areas of the nonwoven material’s surface by an automatic dispenser. Measurements were prepared using an optical microscope M9 No. 63649, lens FMA050 (Moscow, Russia) by Altami studio 3.4 Software. The relative measurement error was ±0.5%

#### 2.2.7. Permeability to Air

Permeability to air characterizes barrier properties of porous nonwoven material. Air permeability of the PHB–Hmi porous samples was measured according to the standard protocol according to Gurley method [61,62]. The pressure was 1.22 MPa, volume of the air was 100 mL, and the test sample’s area was 6.5 cm^2^. The relative measurement error was ±5%

#### 2.2.8. Antimicrobial Tests

The antimicrobial activity of PHB–Hmi samples was studied by biomedical tests on cellular material of *Staphylococcus aureus* p 209, *Salmonella typhimurium* and *Escherichia coli* 1257. Meat-peptone agar was used for cultures of microorganisms, incubation time was 24 h at 37 °C. Concentration of microbial cells in the saline solution was 5 × 10^5^ CFU per mL. The crops were incubated for 48 h at 37 °C after preparation PHB–Hmi samples in Petri dishes with meat–peptone agar. In parallel, the test culture suspensions used in the experiment were seeded to control the concentration of viable microorganisms. The colonies of viable microorganisms grown on the surface of the agar were counted.

#### 2.2.9. Hemin Release Studies

Hemin release study allowed to evaluate time-dependently the amount of additive released from the material. Electrospun materials containing 5% of Hmi (10 × 10 mm^2^) were poured in 1.5 mL of 0.1 M PBS (phosphate-buffered saline, pH 7.4). Samples were incubated under constant shaking of 180 rpm at 37 °C during 48 h. The supernatant samples were picked at 0 h (just after the films soaking), 5 h, 24 h, and 48 h. The released hemin absorbance was determined by UV spectrophotometry (SHIMADZU UV-1800 (Shimadzu, Kyoto, Japan)) at 292 nm. The release data are presented as the average value of five specimens with the standard deviation.

#### 2.2.10. Cell Culture

The immortalized human fibroblasts BJ-5ta cell line was maintained in 25 cm^2^ polystyrene flasks in the DMEM medium supplemented with 10% FBS and gentamycin (50 µg/mL) at 37 °C in a humidified atmosphere containing 5% CO_2_. The cells were replated using trypsin-EDTA solution twice per week.

#### 2.2.11. Cytotoxic Activity Analysis

To assess the cytotoxic activity and biocompatibility, the cells were seeded into 24-well plates (20,000 cells per well) directly before experiment on film samples and incubated under standard conditions for 72 h. Cells photo were taken at 24, 48, 72 h of incubation by Nikon Diaphot phase contrast microscope at 40x magnification and a Levenhuk M1400Plus camera. We applied standard MTT assay to evaluate cells survival [63]. Each well was supplemented with 250 μL of MTT solution (1 mg/mL) in the serum-free DMEM and incubated during 4 h. Next, the medium was aspirated, precipitated formazan crystals in each well were dissolved in 400 μL of DMSO, and the light absorption was measured at 540 nm. Survival curves plotting, IC_50_ values calculation, and statistical analysis were performed in Excel (Microsoft Corporation, Redmond, WA, USA) and OriginPro (version 2020b, OriginLab Corp., Northampton, MA, USA).

## 3. Results and Discussion

### 3.1. Characterization of PHB–Hmi Fibers

**Figure 2:**
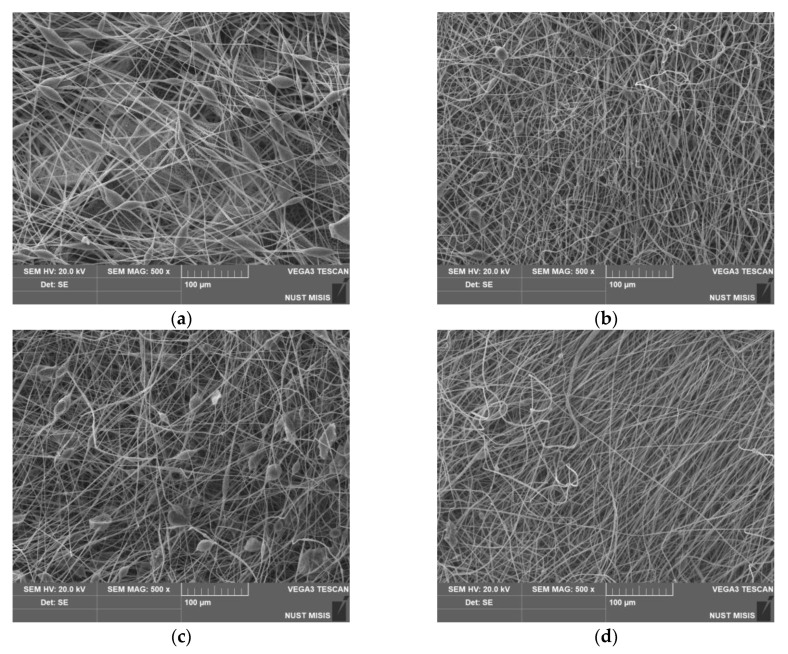
SEM images of electrospun materials based on PHB with different content of Hmi: 0% wt. (**a**), 1% wt. (**b**), 3% wt., (**c**) and 5% wt. (**d**).

Addition of hemin to the poly(3-hydroxybutyrate) fibers is a good approach to modify its surface and properties. The introduction of Hmi into the forming solution increased the electrical conductivity by 10–40% and the viscosity by 40–90%, that contributed to a significant improvement in the fibers’ quality. All key parameters of ES process such as the flow rate of the polymer solution, the curing rate of the fibers, and the trajectory of the thread were more stable due to addition of Hmi to the forming solution. As shown in Figure 2, all the PHB–Hmi fibers displayed uniform and randomly orientated structure. It fully corresponded to the type of the structure produced by the ES method during formation of fibrous layer [64]. One of the important parameters of ES is the distance between the capillary and the collection zone. First of all, this distance affects the size of the ES area, as well as the diameter of the formed fibers, which makes a significant contribution to the formation of a uniform layer of nonwoven material [65]. The optimal distance was selected experimentally taking into account the optimal molding conditions for the PHB solution to obtain a uniform Taylor cone during the molding process [66]. Another significant aspect in the formation of composites by the ES method is the contribution of the solvent. There are a large number of approaches to the implementation of double-solution electrospinning [55,56]. The main contribution of the two-solution ES process of PHB–Hmi composites is due to the fact that PHB is not soluble in N,N-dimethylformamide, and Hmi is not soluble in chloroform. At the same time, the solutions mix well, forming a sufficiently homogeneous system for forming fibrous materials with a uniform distribution of Hmi in the structure [52,53]. Moreover, the introduction of Hmi contributed to changes in the structure of the fibers. Characteristics of the nonwoven materials are presented in Table 3.

The surface density of the material was reduced by 30–40% due to an increase in porosity. The average fibers’ diameter was reduced by 40–50%. With an increase in the concentration of Hmi the number of defects on the surface of the fibers noticeably decreased. Thickenings, gluings, and spherical formations were almost completely absent at 5% wt. of Hmi.

**Figure 3:**
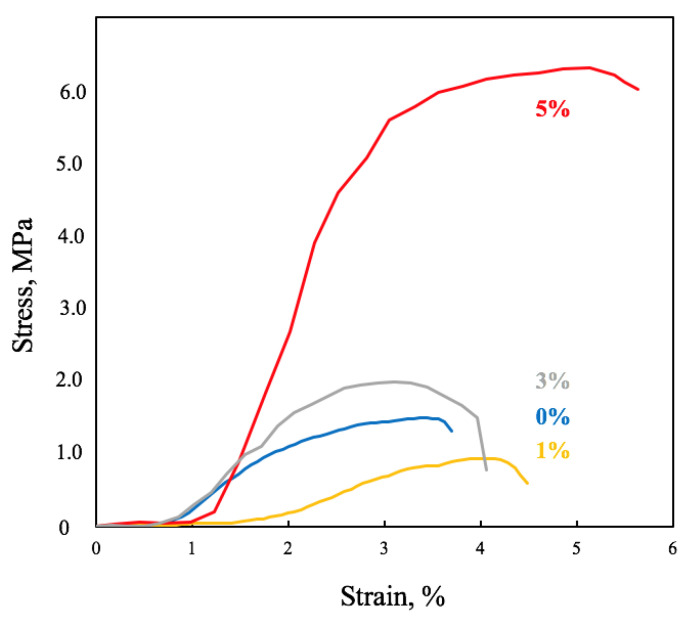
Typical tensile stress-strain curves of electrospun materials based on PHB with different content of Hmi: 0% wt. (**blue**), 1% wt. (**yellow**), 3% wt., (**grey**) and 5% wt. (**red**).

The reduction in the number of defects and the formation of more uniform fibers contributed to the growth of mechanical properties of PHB-based materials. The tensile strength increased by 3.2 times, and the elongation at break increased by 1.7 times. Typical tensile stress−strain curves of electrospun PHB–Hmi materials are shown on the Figure 3. The addition of higher concentrations of Hmi caused weakening of mechanical properties of the material.

**Figure 4:**
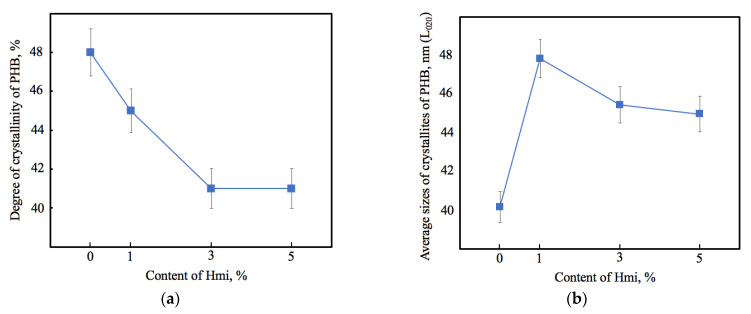
Degree of crystallinity (**a**) and average sizes of PHB crystallites L_020_ (**b**) of PHB–Hmi composites.

The supramolecular structure of the polymer plays a significant role in the key properties of the material including biocompatibility, degradation, stability under different environmental conditions [67]. PHB is a semi-crystalline polymer with the orthorhombic crystal lattice (*a* = 0.576 nm, *b* = 1.320 nm, *c* = 0.596 nm, and space group symmetry of Р2_1_2_1_2_1_) [68]. Hmi did not affect these parameters of native crystalline phase of PHB. However, Hmi significantly affected the degree of crystallinity and the size of the crystallites of PHB (Figure 4). 

The introduction of Hmi led to decrease in the proportion of the crystalline phase by 6–15%, however, the size of the crystallites increased by 26–15%. Probably, hemin could act as a crystallization center during the curing of the forming solution. Thus, PHB was able to form more regular and larger crystallites, which also contributed to the mechanical properties of the material.

These results were consistent with the changes in thermal properties of PHB–Hmi (Table 4). While Hmi very slightly effected on the melting temperature of the crystalline phase, the melting enthalpy varied according to the changes in the degree of crystallinity. During the first melting, it decreased by 12–19%, and during the second one by 13–20%. so slight differences between the first and second heating showed that the polymer in the molding solution had time to crystallize sufficiently, and the fibrous structure had little effect on the phase distribution [69].

These characteristics showed the significant positive contribution of Hmi to the formation of nanomaterials. Exerting a significant influence on the crystallization of PHB, this modifying additive allowed to obtain the material devoid of the disadvantages of pure PHB (PHB–Hmi was more durable, with fewer defects, more uniform fibers). In addition, the significant influence of the Hmi on the molding properties of the solution makes it possible to obtain a material with a more predictable structure, which could not be obtained using other metal-containing modifying additives [53].

### 3.2. The Barrier Properties of PHB–Hmi Fibers

**Figure 5:**
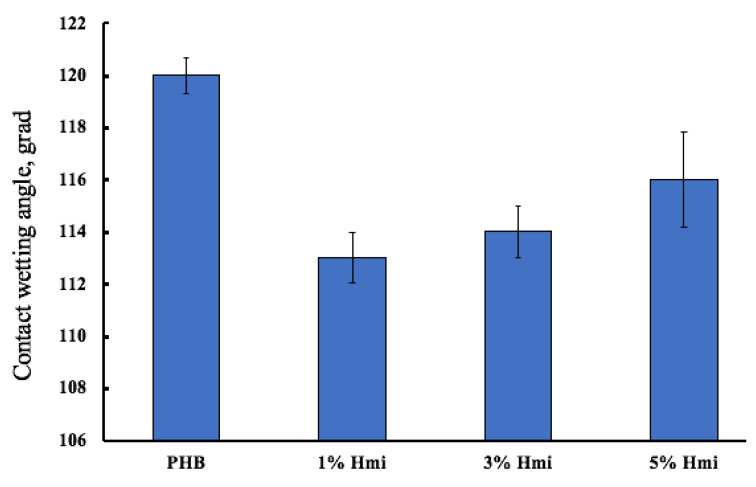
Contact wetting angles of the fibrous materials based on PHB–Hmi.

PHB is a hydrophobic material, which can make it difficult for cells to consolidate in a living organism and slow down the wound healing process. The control of hydrophobicity is an important task. Figure 5 shows the impact of Hmi on the hydrophobicity of the nonwoven material.

The introduction of Hmi has a hydrophilic effect due to the polar groups—COOH (Figure 1b) located in the structure of the tetrapyrrole ring. Moreover, it is known that tetrapyroll complexes tend to mutual aggregation [70], and with increasing the Hmi concentration, this effect could be observed. The wetting angle decreases slightly due to the smaller number of hydrophilic sites that are freely available on the surface of the fibers with the growth of the Hmi concentration.

**Figure 6:**
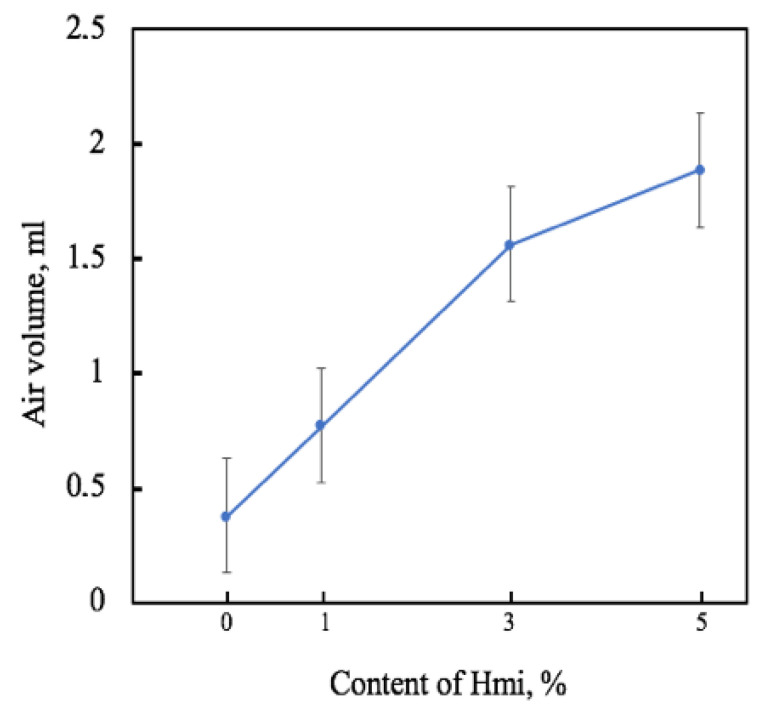
Air volume that passed through the fibrous materials based on PHB–Hmi according to the Gurley method.

Another important aspect of wound healing is the permeability to air [71]. The introduction of Hmi made a significant contribution to the control of the breathability of nonwoven fabric (Figure 6).

The key parameter affecting the permeability of the nanofibrous material is the morphology of the fibrous layer. With an increase in porosity and with a decrease in the number of glues and engagements, the material becomes more accessible for air transfer. Air permeability control is extremely important for the formation of a reliable environment in the wound healing zone. Thus, we observed five-fold air permeability increment with the addition of 5% of Hmi.

### 3.3. The Antimicrobial Tests of PHB–Hmi Fibers

The antimicrobial activity of hemin against *S. aureus* is well known [72]. The results of the antimicrobial efficacy of PHB–Hmi electrospun materials against Gram-positive and Gram-negative cultures are shown in Table 5.

It is known that pure PHB-based materials have no antibacterial activity [73]. On the contrary, PHB is able to be a good substrate in view of its microbiological origin.

Table 5 shows that the increasing of the Hmi concentration leaded to the growth of the antimicrobial activity of the fibrous material. 1% of Hmi leaded to the *S. aureus* CFU decrease by 47%, and of *E. coli* by 90%. In relation to *S. typhimurium*, a small concentration of Hmi was less effective reducing the number of colony-forming units within 15%, and 3 and 5% of Hmi provided high activity against *S. aureus* and *S. typhimurium* inhibiting CFU by 79–89% and 74–75% correspondingly. Moreover, 3 and 5% of Hmi displayed almost 98% CFU inhibition against *E. coli*.

**Figure 7:**
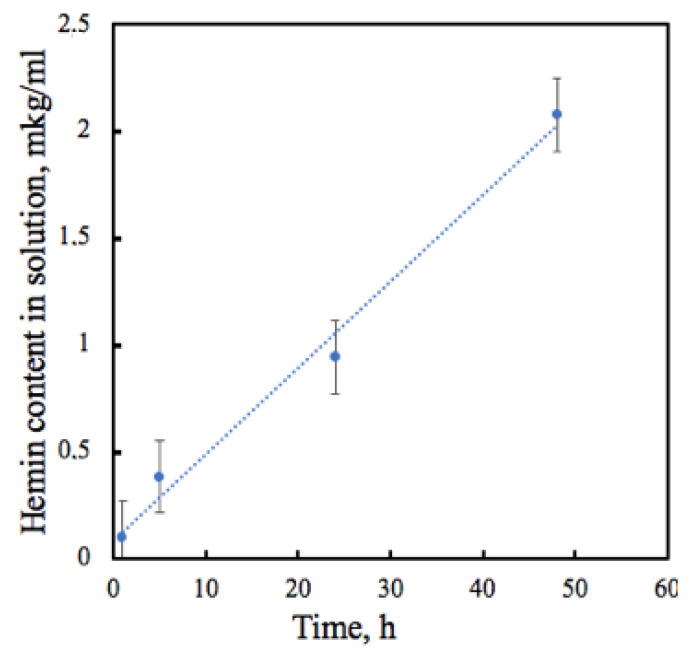
The changes in the Hmi concentration in PBS solution (pH 7.4, 37 °C).

Probably, the antimicrobial effect is explained by the gradual Hmi release from the PHB matrix. The Hmi release profile from the electrospun sample containing 5% of Hmi was recorded from the immersion in PBS solution (pH 7.4, 37 °C) for 48 h. Figure 7 shows the gradual hemin release from PHB containing 5% Hmi. We observed 0.84% Hmi release after 5 h of incubation: 1.72% after 24 h and 4.03% after 48 h. Thus, the released Hmi could explain the average antimicrobial properties of nonwoven materials, which may be beneficial during future applications of these nonwoven materials.

### 3.4. Cytotoxic Activity Analysis and Biocompatibility of PHB–Hmi Fibers

**Figure 8:**
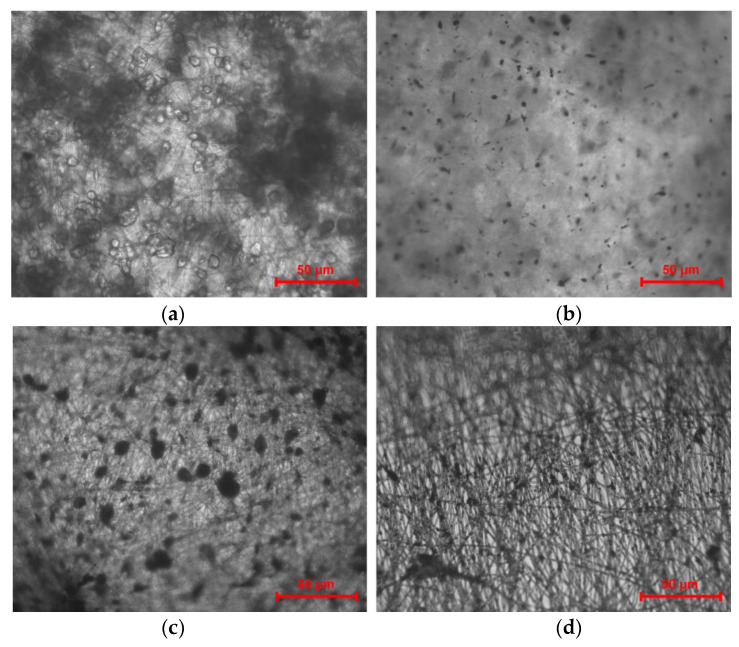
The optical microscopic images of PHB with different hemin content: 0% wt. (**a**), 1% wt. (**b**), 3% wt. (**c**), and 5% wt. (**d**) before fibroblasts seeding BJ-5ta cells. Bar—50 µm.

During microscopic examination, we observed the fibroblasts distribution along the PHB-based fiber samples after 24, 48 and 72 h of the cultivation. The microscopic images of control PHB with different hemin content in cells-free DMEM are represented in Figure 8.

**Figure 9:**
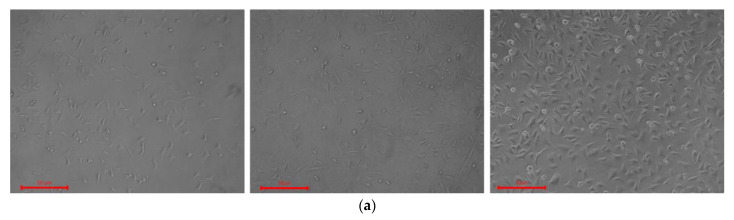

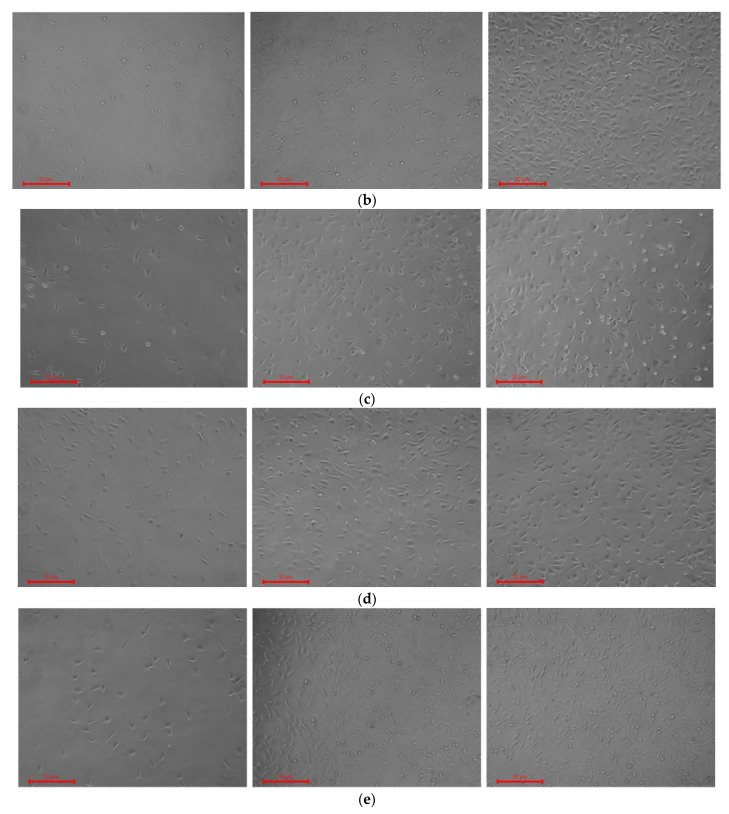
The optical microscopic images of the fibroblasts BJ-5ta after 24, 48 and 72 h (the images aligned left to right respectively) of cultivation in presence of PHB with different content of the hemin: 0% wt. (**b**), 1% wt. (**c**), 3% wt. (**d**), and 5% wt. (**e**); (**a**) control. Bar—50 µm.

The cells exhibited a flattened morphology and demonstrated a good adherence to the polymeric plate surface in presence of PHB membranes. The normal morphology and proliferation rates were comparable with control cells (Figure 9a) evidencing the lack of noticeable cytotoxic effect of the PHB-based fibers and good potential biocompatibility.

According to Figure 9, the cells revealed high viability after 24–72 h of incubation in presence of PHB with different hemin content, indicating low PHB toxicity.

**Figure 10:**
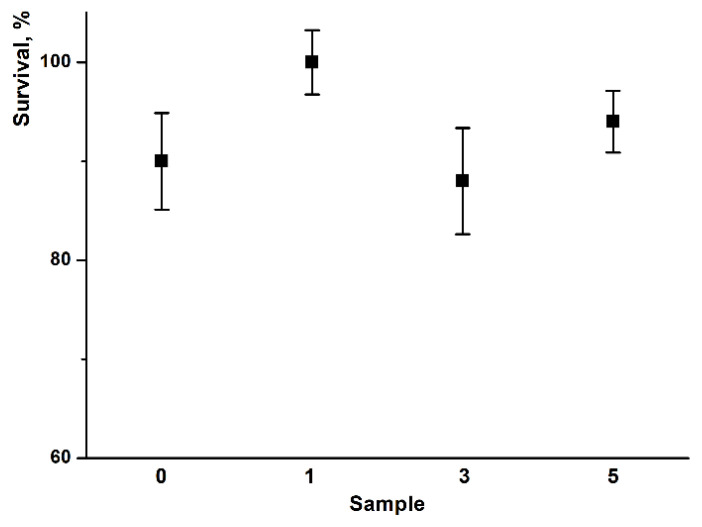
Survival of BJ-5ta cells after 72 h incubation with the PHB with different content of the hemin (0—0% wt., 1—1% wt., 3—3% wt., and 5—5% wt). (mean ± SD, *n* = 3).

The MTT test results (Figure 10) also evidenced the absence of pronounced cytotoxic effects of PHB-based fibers.

Summarizing, there were no significant differences in morphology, cells shape, adherence, or survival rate between groups after 24, 48, and 72 h of incubation of BJ-5ta cells with PHB with different hemin content (Figure 9 and Figure 10). The lack of ruptures, deformations, or other phenomena confirmed the good biocompatibility of the examined PHBs. Good biocompatibility is one of the basics of the materials applied in medicine. Thus, these preliminary results evidence that all examined PHB-based fibers lack toxic effects on cell viability and morphology.

## 4. Conclusions

We evaluated the effect of 1–5% wt. of the hemin molecular complexes on the structure and properties of the composite materials based on poly-3-hydroxybutyrate in this research. The Hmi made it possible to obtain fibers with improved morphology. The presence of an iron atom in the Hmi structure significantly improved the properties of the solution during the ES process, which had a positive effect on the structural characteristics of the material. As a result, the tensile strength of the fibrous layer increased by 3.2 times, and the elongation at break increased by 1.7 times with the introduction of 5% wt. of Hmi. Although it should be mentioned that the high modulus of elasticity of the samples, which may impose restrictions on the use of film samples in clinical products, however, electrospun nonwoven samples are characterized by high softness and elasticity, due to the high degree of freedom of the fibers relative to each other. Moreover, Hmi acted as a crystallization center, which allowed the formation of a more favorable crystal structure of the polymer. The size of PHB crystallites increased, and their total fraction decreased. The Hmi antimicrobial activity ensured the death of both Gram-negative and Gram-positive cultures after contact with the PHB–Hmi fibrous material. Cytotoxic activity results demonstrated that formulated PHB-based fibers characterized with high potential safety and could be promising vehicles for regenerative medicine applications. Despite the low statistical differences in the MTT results, we could assume influence of the localization and behavior of Hmi in the material on the cells survival. As mentioned earlier, tetrapyrroles are prone to aggregation processes which are directly related to their concentration [70,74]. The minimum of the aggregation was detected at 1% of Hmi and corresponded to a high survival rate. The maximum of aggregation was detected at 3% of Hmi and corresponded to a low survival rate, while at 5% of Hmi, there were both aggregated and free components. This assumption is consistent with the previously described trends in the accumulation of iron atoms obtained by the EDX atomic analysis of an iron atom [49]. Thus, we can assume presence of dependence between Hmi aggregation degree and cells survival, what, of course, have to be tested further. Thus, summarizing the data obtained, we can recommend PHB–Hmi materials for regenerative medicine as wound dressing layers, and among alternative applications, we can offer hygienic agents, filter materials, and other clinical products that require a highly developed surface in combination with antimicrobial properties and biocompatibility. 

## Figures and Tables

**Table 1:** Conditions of the ES method.

Diameter of Capillary, mm	Voltage, kV	Distance between the Electrodes, mm	Gas Pressure on the Solution, kg(f)/cm^2^
0.1	17–20	190–200	10–14

**Table 2:** The properties of the forming solution.

Content PHB, wt. %	Content of Hmi, wt. % of PHB Mass	Electrical Conductivity, μS/cm	Viscosity, Pa s
7	0	10	1.0
7	1	11	1.4
7	3	13	1.7
7	5	14	1.9

**Table 3:** Material properties of PHB–Hmi fibers ^a^.

Sample	Density, g/cm^3^ (Mean ± SD, *n* = 10)	Average Diameter, µm (Mean ± SD, *n* = 100)	Tensile Strength, MPa (Mean ± SD = 0.05, *n* = 10)	Elongation at Break, % (Mean ± SD = 0.2, *n* = 10)
PHB 0% wt.	0.30 ± 0.01	3.50 ± 0.08	1.7	3.6
PHB with 1% wt. of Hmi	0.20 ± 0.02	2.06 ± 0.07	0.7	4.7
PHB with 3% wt. of Hmi	0.20 ± 0.01	1.77 ± 0.04	1.9	4.7
PHB with 5% wt. Hmi	0.17 ± 0.01	1.77 ± 0.04	5.5	6.1

^a^ Density and average diameter were calculated per area 400 × 600 µm^2^.

**Table 4:** Thermal properties of PHB–Hmi, where χ—crystallinity degree Δ ± 2.5%, ∆H—melting enthalpy Δ ± 2.5%, and T_m_—melting temperature Δ ± 2%.

Sample	Concentration of Additive, %	First Heating Run		Second Heating Run	
		T_m_, °C	ΔH, J/g	T_m_, °C	ΔH, J/g
PHB	0	175	93.1	170	90.8
PHB–Hmi	1	172	81.8	168	78.7
PHB–Hmi	3	173	77.8	170	75.4
PHB–Hmi	5	174	75.3	170	72.7

**Table 5:** Antimicrobial activity of electrospun fibrous materials based on PHB–Hmi composites.

Test Culture	Initial Test Culture, CFU/mL	Sample, CFU/mL	Control, CFU/mL
	PHB with 1 % wt. Hmi		
*S. aureus* р 209	2.0 × 10^4^	4.5 × 10^3^	8.6 × 10^3^
*E. coli* 1257	2.0 × 10^4^	8.5 × 10^2^	9.8 × 10^3^
*S. typhimurium*	2.0 × 10^4^	7.2 × 10^3^	8.1 × 10^3^
	PHB with 3 % wt. Hmi		
*S. aureus* р 209	2.1 × 10^4^	1.8 × 10^3^	8.6 × 10^3^
*E. coli* 1257	2.0 × 10^4^	<1 × 10^2^	9.8 × 10^3^
*S. typhimurium*	2.0 × 10^4^	2.1 × 10^3^	8.1 × 10^3^
		PHB with 5 % wt. Hmi	
*S. aureus* р 209	2.0 × 10^4^	0.9 × 10^3^	8.6 × 10^3^
*E. coli* 1257	2.0 × 10^4^	<1 × 10^2^	9.8 × 10^3^
*S. typhimurium*	2.0 × 10^4^	2.0 × 10^3^	8.1 × 10^3^

## Data Availability

Data are contained within the article.
